# Alterations in metabolome and microbiome signatures provide clues to the role of antimicrobial peptide KT2 in ulcerative colitis

**DOI:** 10.3389/fmicb.2023.1027658

**Published:** 2023-02-09

**Authors:** Qiong Nan, Yan Ye, Yan Tao, Xinyi Jiang, Yinglei Miao, Jie Jia, Jiarong Miao

**Affiliations:** ^1^Department of Gastroenterology, First Affiliated Hospital of Kunming Medical University, Kunming, Yunnan, China; ^2^Yunnan Province Clinical Research Center for Digestive Diseases, First Affiliated Hospital of Kunming Medical University, Kunming, Yunnan, China; ^3^Scientific Research Laboratory Center, First Affiliated Hospital of Kunming Medical University, Kunming, Yunnan Province, China

**Keywords:** ulcerative colitis, gut, microbiome, metabolomics, biomarkers

## Abstract

**Introduction:**

Ulcerative colitis (UC) is an inflammatory disease of the intestinal tract with unknown etiology. Both genetic and environmental factors are involved in the occurrence and development of UC. Understanding changes in the microbiome and metabolome of the intestinal tract is crucial for the clinical management and treatment of UC.

**Methods:**

Here, we performed metabolomic and metagenomic profiling of fecal samples from healthy control mice (HC group), DSS (Dextran Sulfate Sodium Salt) -induced UC mice (DSS group), and KT2-treated UC mice (KT2 group).

**Results and Discussion:**

In total, 51 metabolites were identified after UC induction, enriched in phenylalanine metabolism, while 27 metabolites were identified after KT2 treatment, enriched in histidine metabolism and bile acid biosynthesis. Fecal microbiome analysis revealed significant differences in nine bacterial species associated with the course of UC, including *Bacteroides*, *Odoribacter*, and *Burkholderiales,* which were correlated with aggravated UC, and *Anaerotruncus*, *Lachnospiraceae*, which were correlated with alleviated UC. We also identified a disease-associated network connecting the above bacterial species with UC-associated metabolites, including palmitoyl sphingomyelin, deoxycholic acid, biliverdin, and palmitoleic acid. In conclusion, our results indicated that *Anaerotruncus*, *Lachnospiraceae*, and *Mucispirillum* were protective species against DSS-induced UC in mice. The fecal microbiomes and metabolomes differed significantly among the UC mice and KT2-treated and healthy-control mice, providing potential evidence for the discovery of biomarkers of UC.

## 1. Introduction

The incidence of inflammatory bowel disease (IBD) ulcerative colitis (UC) has increased rapidly in China in recent years (Ng et al., 2013). Complex genetic and environmental factors are involved in the etiology of UC and may be related to epithelial barrier function and immune response with genetic susceptibility (Ordas et al., 2012).

Changes in the intestinal microbiota are a crucial environmental factor in the development of IBD (Xavier and Podolsky, 2007). Animal models are important for studying the intestinal microbiota of UC patients and elucidating the underlying pathogenesis. Notably, changes in intestinal microbial composition are often reported in UC patients (Takeshita et al., 2016; Imhann et al., 2018; Walujkar et al., 2018). As gut microbiota can influence host metabolites (Wan et al., 2022), clarifying the changes in microbiota and metabolites in the gut of UC patients is essential.

Antimicrobial peptides (AMPs) are short amino acids with innate immune defense functions (Tornesello et al., 2020) that regulate the composition of the intestinal microbiome, thereby altering the relationships between the microbiota and intestinal barrier in UC patients (Gubatan et al., 2021), suggesting great potential in disease treatment.

KT2 is a cationic, amphipathic, ultra-short AMP, which shows antibacterial activity against both gram-negative and gram-positive bacteria but low toxicity toward normal cells (Anunthawan et al., 2013). We previously found that KT2 can restrain Th17 cell differentiation in UC models and slow UC progression (Gu et al., 2022). However, the detailed mechanism by which KT2 regulates UC development requires further study.

We hypothesized that KT2 alters the composition of the intestinal microbiota, and thus the metabolites in UC patients. Therefore, in the present study, we characterized the changes in the intestinal microbiota and metabolites in UC mice with and without KT2 treatment. The bacterial abundances of several species, including *Bacteroides*, *Odoribacter*, *Anaerotruncus*, and *Lachnospiraceae*, were similar between the heathy control (HC) and KT2-treated UC mice, but not with the non-treated UC mice. This study identified several metabolites as potential biomarkers for UC, which may help guide the application of specific probiotics in UC treatment.

## 2. Materials and methods

### 2.1. Construction of a mouse model of UC

Twelve C57BL/6 male mice (6–8 weeks old, weighing 18–22g) were bought from the Animal Experiment Center of Kunming Medical University (Kunming Medical University Animal Experiment Center, Kunming, China). All mice were randomly divided into three groups and kept in cages at 25 ± 2°C. To avoid cage and maternal effects on the gut microbiome (Singh et al., 2021), the mice were fed separately under clean conditions (SPF Animal Experimental Center of Kunming Medical University). Each mouse was housed in an independent environment, with no inter-mouse interactions. The mice were given free access to water and food and were maintained under 12 h:12 h light: dark cycle. The pre-experiment period lasted 1 week.

The KT2 peptide (purity >95%) was provided by Nanjing Jiepeptide Biotechnology Co., Ltd. DSS was provided by the MP Corporation (USA). The KT2 was prepared as 2 mg/mL solution in phosphate-buffered saline (PBS) and stored at −80°C. The UC model was established according to prior research (Lv et al., 2018) and KT2 treatment was administered following previous study (Maraming et al., 2018), The disease activity index (DAI) was determined (Xu et al., 2017; Yan et al., 2022). The mice were sacrificed with pelltobarbitalum natricum on 10 d, and their colons were surgically removed. Colon length was measured, and histological scores were obtained to assess tissue damage.

All animal assays were conducted in accordance with the Guide for the Care and Use of Laboratory Animals and were approved by the Animal Ethics Committee of Kunming Medical University, China (No. kmmu20211575).

### 2.2. Metagenomic DNA extraction and sequencing

Each fecal sample (200 mg) from HC, UC and KT2 groups was suspended in 250 μl of guanidine thiocyanate with 0.1 M Tris (pH 7.5) and 40 μl of 10% N-lauroyl sarcosine. DNA was extracted using a Qiagen QIAamp DNA Stool Mini Kit (Qiagen, Germany). DNA concentration was estimated using a NanoDrop instrument (Thermo Scientific, Wilmington, DE), and DNA molecular weight was estimated by agarose gel electrophoresis.

A DNA library was constructed according to previous study (Nurk et al., 2017). Paired-end metagenomic sequencing (2 × 150 base pairs) was performed on the Illumina platform.

### 2.3. Taxonomic annotation

Clean reads of metagenome sequencing were processed with MetaPhlAn2 (Segata et al., 2012) to obtain taxonomic profiles from a database of clade-specific marker genes. In total, one million unique clade-specific marker genes were identified.

### 2.4. Metabolite extraction, profiling, and analysis

Metabolites were profiled in fecal samples. Briefly, the fecal samples (50 mg) were completely homogenized in a 2-mL tube, followed by the addition of 800 μL of 80% methanol. After vortexing for 90 s at 65 Hz, the mixture was ultrasonically treated for 30 min, then maintained at −20°C for 1 h. The mixture was then centrifuged at 12,000 rpm for 15 min at 4°C. The resulting supernatant (1,200 μL) was filtered through a 0.22 μM membrane, with the filtrate (200 μL) then mixed with 5 μL of lysophosphatidylcholine (LPC; 12:0; 0.14 mg/mL) and transferred to a 1.5-mL microcentrifuge tube.

Ultra-performance liquid chromatography–tandem mass spectrometry (UPLC-MS/MS) was performed with a Waters ACQUITY UPLC Scientific Q-Exactive high-resolution mass spectrometer (Thermo Scientific, Wilmington, DE) and an ACQUITY UPLC HSS T3 column (2.1 × 100 mm and 1.7 μm). The column temperature was set to 40°C and the flow rate was set to 0.3 mL/min. Mobile phase A consisted of water and 0.05% formic acid, and mobile phase B consisted of acetonitrile. The injection volume was 6 μL at 4°C.

The positive-ion conditions were as follows: Heater temperature, 300°C; sheath gas flow rate, 45 arbs; aux gas flow rate, 15 arbs; sweep gas flow rate, 1 arb; spray voltage, 3.0 kV; capillary temperature, 350°C; and S-lens RF level, 30%. The negative-ion conditions were as follows: Heater temperature, sheath gas flow rate, aux gas flow rate, sweep gas flow rate, and capillary temperature consistent with positive-ion conditions; spray voltage, 3.2 kV; and S-lens RF level, 60%. The MS analysis was performed in full-scan dd-MS2 (TopN = 10) mode with a scan range of 70 to 1,000 m/z.

### 2.5. Differential analysis of metabolites in HC, DSS, and KT2-treated groups

Multivariate analysis was used to analyze differences in metabolites among the three groups. Differential metabolites (DMs) were selected based on *p* < 0.05 (Student’s *t*-test), log_2_FC > 1, and Variable Importance in Projection (VIP) > 1.

### 2.6. Hierarchical cluster analysis of DMs

To investigate the similarity in DMs functions, we performed hierarchical cluster analysis using complete-linkage and counting the Euclidean distance matrix. The results were visualized as a heatmap.

### 2.7. Functional analysis of DMs

MetaboAnalyst^1^ was used for metabolic pathway analysis using the Kyoto Encyclopedia of Genes and Genomes (KEGG) library.

### 2.8. Correlation analysis of DMs and differential microbes

Correlations between DMs and differential microbes were analyzed by calculating Pearson correlation coefficients. Correlations with Spearman’s *r* > 0.05 and *p* < 0.05 were considered statistically significant.

### 2.9. Hematoxylin–eosin (H&E) staining

Colon tissue samples were kept in 10% formaldehyde (Sigma-Aldrich, Missouri, USA) for 1 d. After dehydration and paraffin infiltration, the tissues were embedded in paraffin and cut into 5-μm thick sections. The slices were then stained with H&E (Solarbio, Beijing, China) and imaged under a light microscope (Olympus, Tokyo, Japan) for histological evaluation based on previously described parameters (Kihara et al., 2003).

### 2.10. Enzyme-linked immunosorbent assay (ELISA) for detection of inflammatory cytokines

Transforming growth factor β (TGF-β) concentrations in the colon calprotectin (FC) concentrations in the feces were detected using ELISA kits (China Enzyme Immunoassay Co., Ltd) in accordance with the manufacturer’s instructions. Optical density (OD) was measured at 450 nm and the corresponding concentration was calculated.

### 2.11. Cytokine mRNA expression detection by real-time polymerase chain reaction (RT-PCR)

RNA-Easy™ Isolation Reagent (Vazyme Biotech Co., Ltd., Nanjing, China) was used to extract RNA from the colon. A Maxima First-Strand cDNA Synthesis Kit (Thermo Scientific, USA) was used for reverse transcription synthesis of cDNA. The mRNA level of individual genes was measured by RT-PCR with a fluorescent quantitative reagent. The relative expression levels of inflammation-related genes Tumor necrosis factor ligand member 2 (*TNF-α*), Interleukin-6 (*IL-6*), and Interleukin-1 beta (*IL-1β*) in colon tissues were analyzed by comparing threshold period (Ct) analysis of the data with *GAPDH* as an internal reference.

### 2.12. Statistical analysis

Data were expressed as mean ± standard error of the mean (SEM). Spearman correlation analysis was performed between microbiota and metabolites. Significant differences were defined at *p* < 0.05. Student’s *t*-tests were performed and adjusted using the Benjamini-Hochberg correction. All data were analyzed using GraphPad Prism 8.4.

## 3. Results

### 3.1. Alleviation of UC symptoms in mice following KT2 treatment

**Figure 1:**
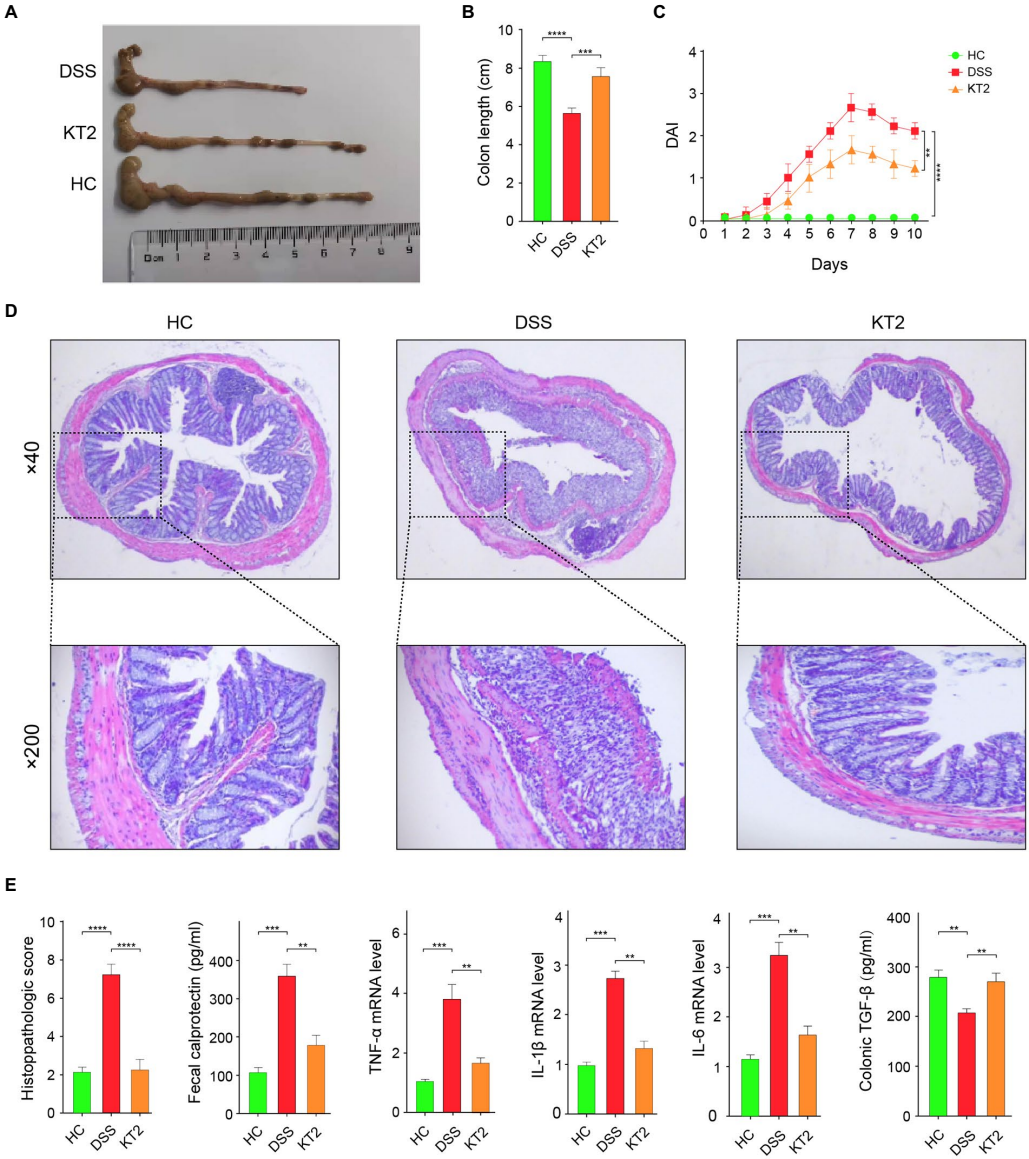
Protective effects of KT2 peptide on UC in mice. **(A)** Colon length in different groups. **(B)** Statistical analysis of colon length in different groups. **(C)** Statistical analysis of DAI scores. **(D)** Pathological changes in colon tissue were assessed using H&E staining. Upper panel shows mouse colon pathology at 40×, lower panel shows mouse colon pathology at 200×. **(E)** Histopathological score and fecal calprotectin, *TNF-α*, *IL-6*, and *IL-1β* levels examined by quantitative RT-PCR (qRT-PCR), and colon TGF-β level examined by ELISA, from left to right. ^∗^*p* < 0.05, ^∗∗^*p <* 0.01, and ^∗∗∗^*p* < 0.001.

We demonstrated that the damaged colon symptoms in the DSS-induced UC mice were alleviated by KT2 treatment, as displayed in Figure 1. Notably, colon length decreased in the DSS group compared with the HC group but increased in the KT2 group (Figures 1A,B). Furthermore, DAI scores increased in the DSS group, but were markedly reversed in the KT2-treated group (Figure 1C). The H&E staining results showed normal colon structure and intestinal crypt morphology in the HC group. In contrast, the DSS group showed structural damage to the colon, with characteristic mucosal erosion, ulcer formation, and high inflammatory cell infiltration. After KT2 treatment, however, inflammation was significantly reduced, damaged mucosa was repaired and regenerated, goblet cell number and crypts were increased, and inflammatory cell infiltration was reduced (Figure 1D). Colon histological scores were markedly elevated in the DSS group compared to the HC group but were notably reduced in the KT2-treated group. Furthermore, TGF-β expression was significantly decreased in the DSS group but increased in the KT2-treated group. The levels of *TNF-α*, *IL-6*, *IL-1β*, and fecal calprotectin were increased in the DSS group but decreased in the KT2-treated group to levels similar to those in the HC group (Figure 1E). These results suggest that KT2 can alleviate DSS-induced UC symptoms.

### 3.2. Changes in bacterial diversity in fecal microbiota associated with UC

**Figure 2:**
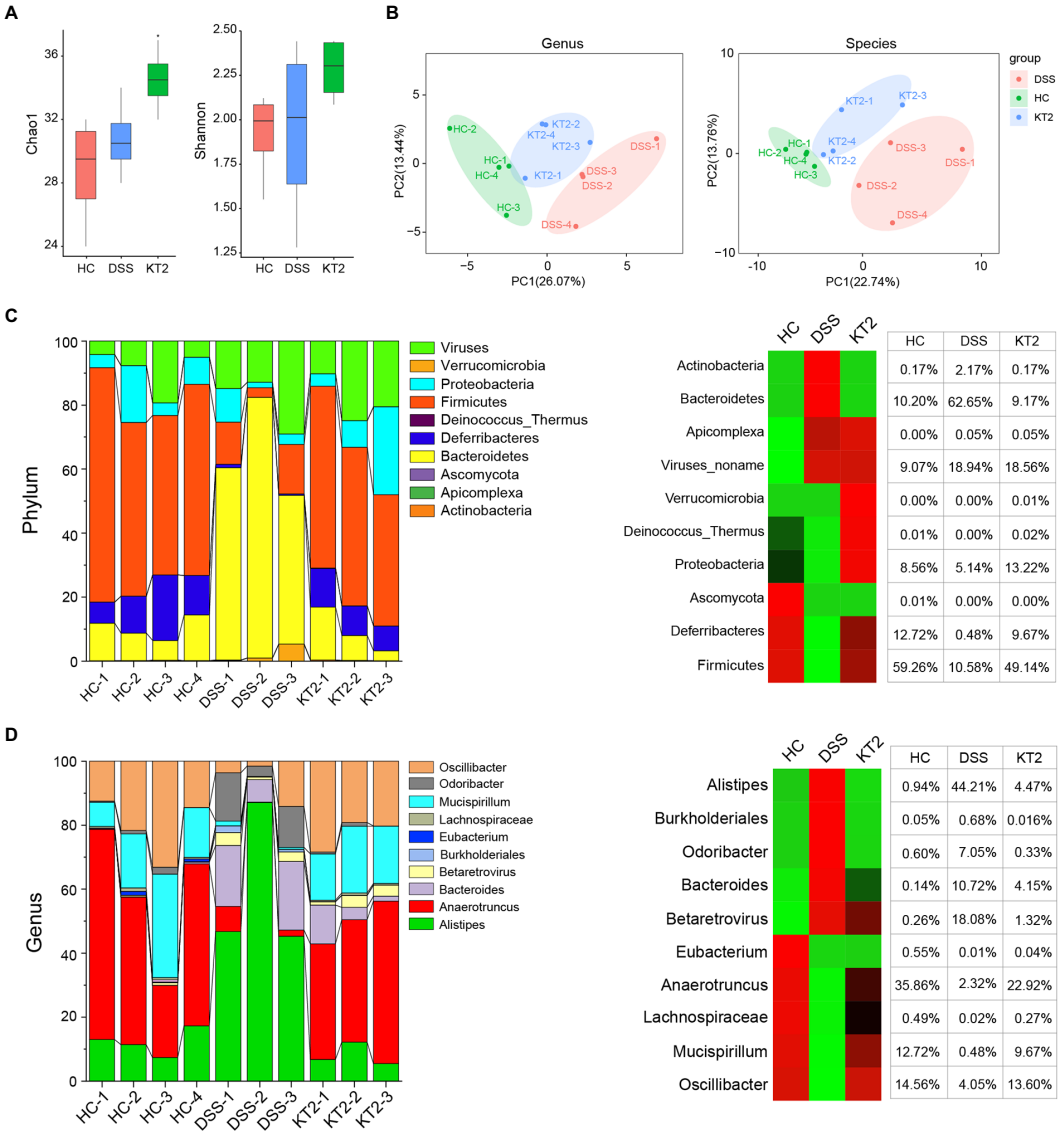
R1. Gut microbiome diversity and structural analysis. **(A)** Species diversity differences in HC, DSS, and KT2 groups, estimated by observed species and Shannon and Chao 1 indices. **(B)** PCA plot based on relative abundance of species showing bacterial structural clustering. (i) Left panel shows PCA plot between HC and DSS groups, (ii) right panel shows PCA plot between the DSS and KT2 groups. Dots represent individual samples. **(C)** Component proportion of bacterial phyla in each group (top 10). **(D)** Component proportion of bacterial genera in each group (top 10).

The Shannon and Chao 1 indices were estimate to evaluate bacterial diversity in different groups. Results showed significant differences in the Chao 1 index between the HC and KT2 groups (*p* < 0.05), but no significant differences between the DSS and KT2-treated groups. The Shannon and Chao 1 indices were similar in the HC, DSS, and KT2 groups. There was no significant differences in the Shannon indices among the HC, DSS, and KT2 groups (Figure 2A). The principal component analysis (PCA) showed separation among the groups (Figure 2B). Therefore, these results suggest that intestinal microbiota diversity was strongly influenced by UC.

The proportions of dominant taxa at the phylum and genus level were assessed among the different groups. We observed considerable variation in the gut microbiota of each group. The top 10 differentially enriched phyla and genera in the different groups were identified (Figures 2C,D). *Anaerotruncus* (genus) and *Firmicutes* (phyla) were dominant in the HC (35.86% and 59.26%, respectively) and KT2 (22.92% and 49.19%, respectively) groups. However, in the DSS group, Anaerotruncus and Firmicutes abundance was low (2.32% and 10.58%, respectively), with *Bacteroidetes* (phyla) and *Alistipes* (genus) found to be most dominant (62.65% and 44.21%, respectively). In contrast, the HC and KT2 groups showed a low abundance of *Bacteroidetes* (10.20% and 9.17%, respectively) and *Alistipes* (0.94 and 4.47%, respectively).

**Figure 3:**
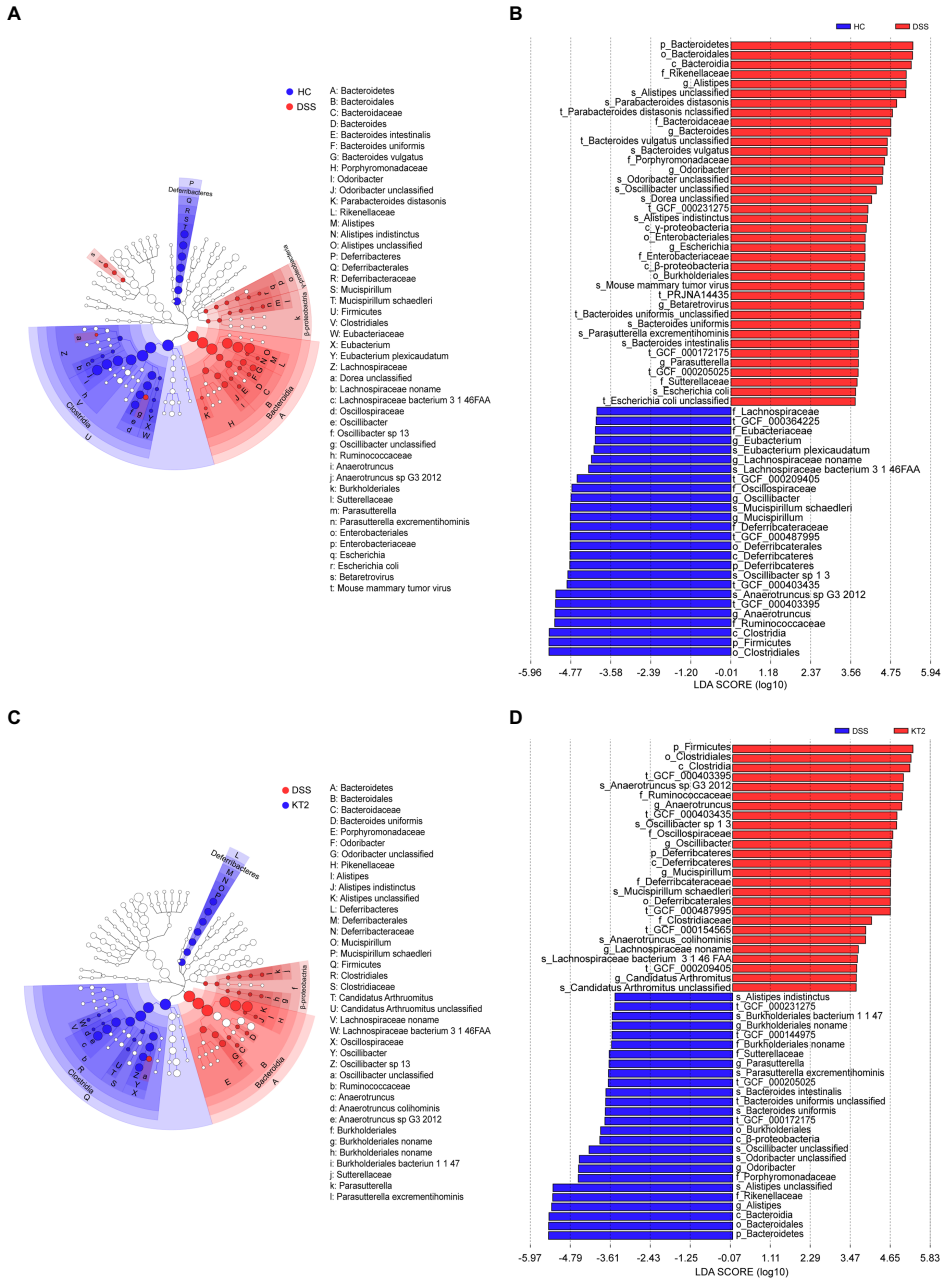
Linear discriminant analysis (LDA) effect size (LEfSe). **(A, B)** Cladogram indicating phylogenetic distribution of microbiota correlated with DSS and KT2 groups. **(C, D)** Differences in abundance between the DSS and KT2 groups.

Linear discriminant analysis (LDA) effect size (LEfSe) was used to generate a cladogram to identify specific microbes associated with DSS and KT2 treatment (Figure 3). *Lachnospiraceae*, *Ruminococcaceae*, *Oscillospiraceae*, *Eubacteriaceae*, and *Deferribacteraceae* were the most abundant microbiota in the HC group, with LDA scores (log10) > 3.6, whereas *Porphyromonadaceae*, *Rikenellaceae, Bacteroidaceae*, *Sutterellaceae*, and *Enterobacteriaceae* were significantly over-represented in the DSS group, with LDA scores (log10) > 3.5 (Figures 3A,B). Similar to the HC group, *Lachnospiraceae, Ruminococcaceae, Clostridiaceae, Oscillospiraceae,* and *Deferribacteraceae* were significantly over-represented in the KT2 group, with LDA scores (log10) > 3.5 (Figures 3C,D). LEfSe analysis of specific microbes showed similarity in microbes in HC versus DSS and DSS versus KT2 (Supplementary Figure S1). These data indicate that intestinal microbes are significantly associated with the course of UC, and differentially abundant microbes can differentiate the microbiota of HC and DSS mice. Furthermore, KT2 administration can modulate abnormal intestinal microbes in UC mice.

### 3.3. Gut metabolome in fecal samples in HC, DSS, and KT2 groups

**Figure 4:**
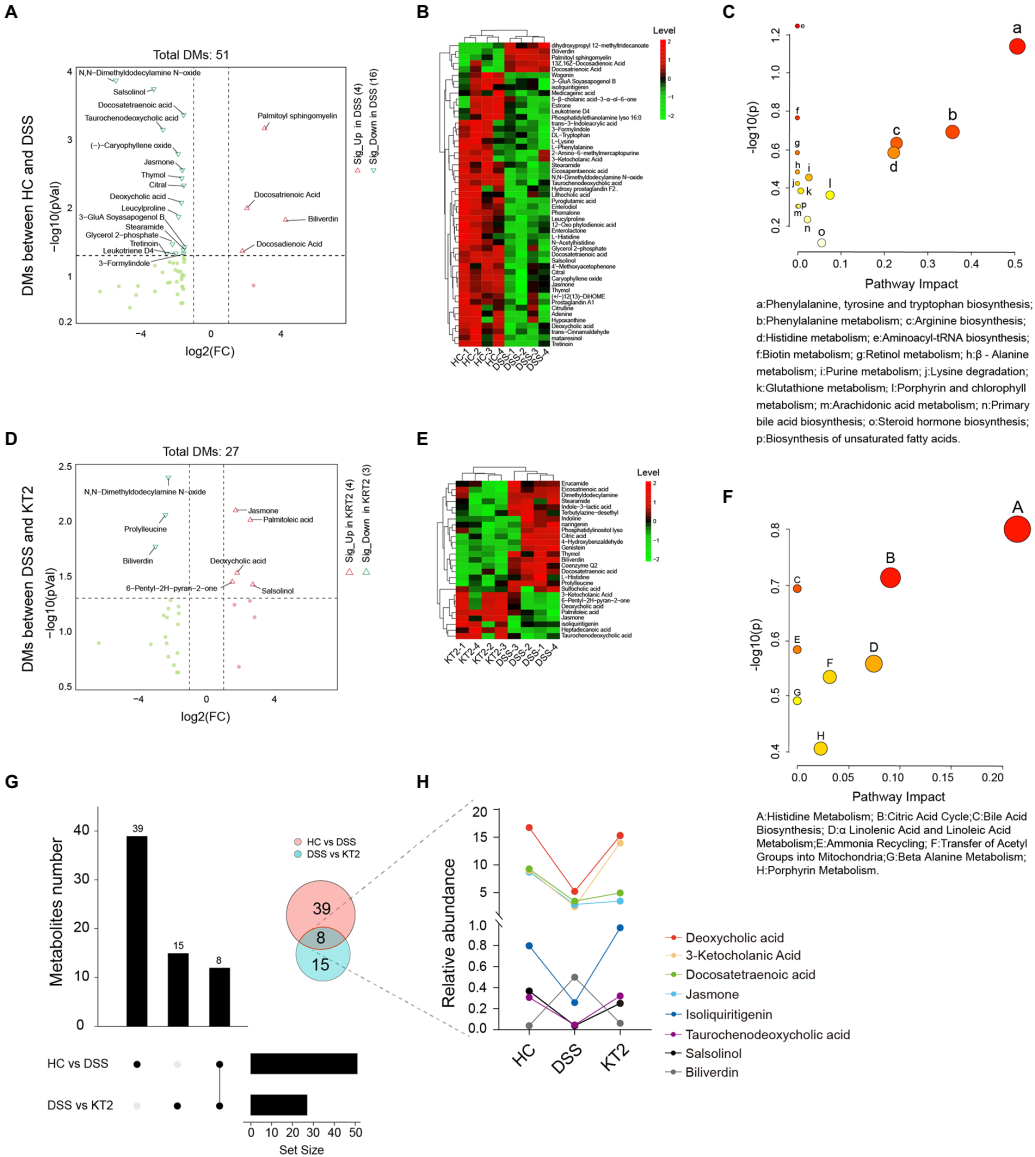
Fecal metabolomics for quantification of metabolites in both HC and DSS groups. **(A)** Volcano plot showing differentially accumulated [log_2_ (FC) on X-axis] and significantly changed [−log_10_ (*p*) on Y-axis] metabolites in HC and DSS groups. **(B)** Hierarchical cluster analysis of metabolites in the HC and DSS groups based on z-normalized abundances. **(C)** Pathway enrichment and significance of DMs between HC and DSS groups. **(D)** Volcano plot showing differentially accumulated [log_2_ (FC) on X-axis] and significantly changed [−log_10_ (*p*) on Y-axis] metabolites in DSS and KT2 groups. **(E)** Hierarchical cluster analysis of metabolites in DSS and KT2 groups based on z-normalized abundances. **(F)** Pathway enrichment and significance of DMs between DSS and KT2 groups. **(G)** Venn diagram of common DMs in HC and DSS groups, in the DSS and KT2 groups. **(H)** DM trends in different groups.

Given the association between intestinal microbes and UC, we performed metabolomic analysis of fecal samples. The differentially changed metabolites in the HC, DSS, and KT2 groups were shown in Figure 4. Results indicated that the salsolinol, jasmone, and deoxycholic acid metabolites were significantly down-regulated in the DSS group (Figure 4A), but significantly up-regulated in the KT2 group. Furthermore, DMs down-regulated in the DSS group (Figure 4B) were enriched in the phenylalanine metabolism, arginine biosynthesis, and histidine metabolism pathways (Figure 4C). Only a few DMs were identified after KT2 treatment, which were enriched in metabolic-related pathways such as histidine metabolism, citric acid cycle, and bile acid biosynthesis (Figures 4D,F). DMs between the HC and KT2 groups were enriched in histidine metabolism, sphingolipid metabolism, and TCA cycle (Supplementary Figure S2).

We also identified several metabolites changed in both the DSS and KT2 groups (Figure 4G), which may be related to the course of UC. For instance, deoxycholic acid, 3-ketocholanic acid, isoliquiritigenin, taurochenodeoxycholic acid, and salsolinol metabolites were significantly decreased in the DSS group but significantly increased in the KT2 group to levels close to the HC group (Figure 4H). Taken together, these results indicate that a specific intestinal metabolome exists in UC mice and that KT2 administration can adjust aberrant intestinal metabolites in UC mice.

### 3.4. Integration of metagenomes and metabolomes

**Figure 5:**
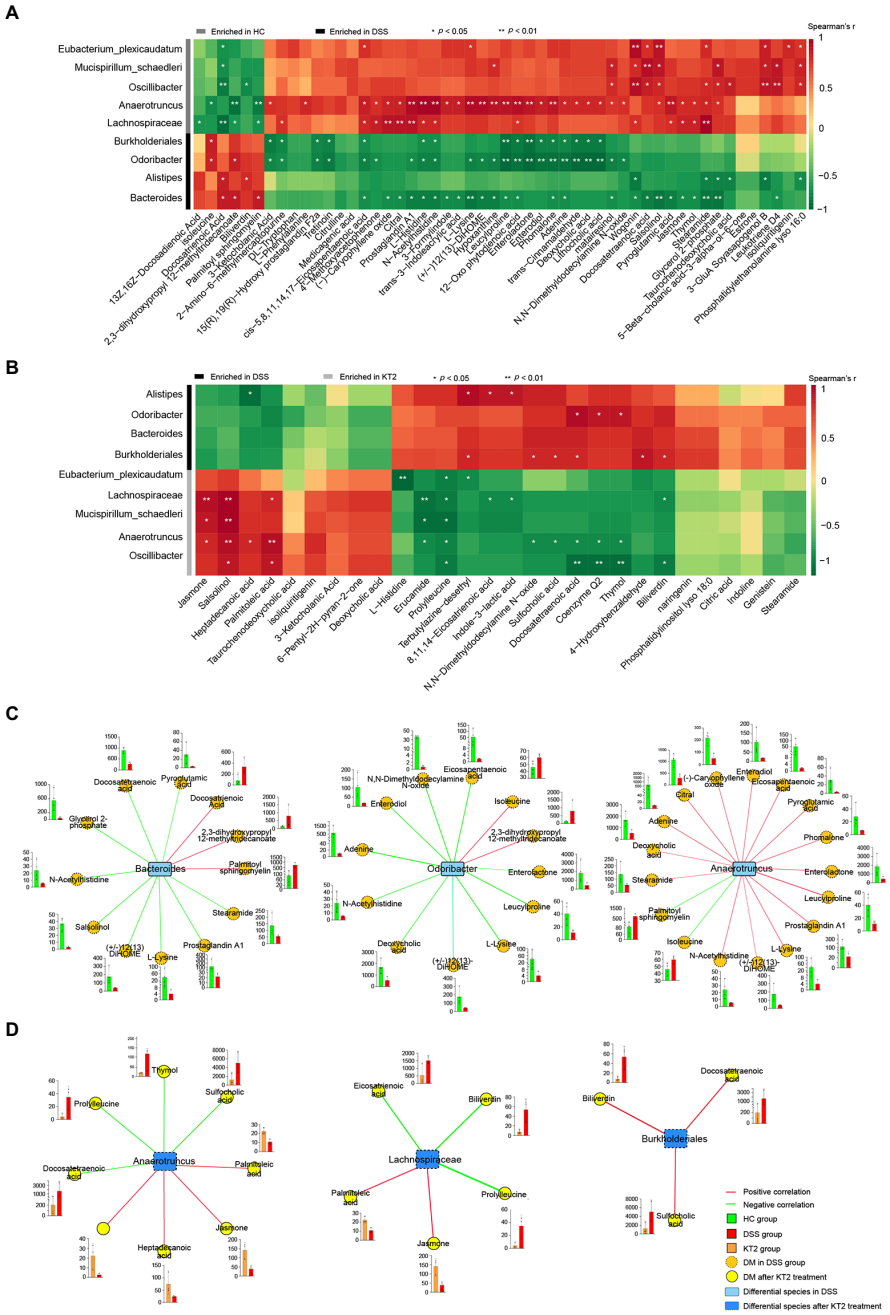
Integration of microbiomes and metabolomes. **(A)** Associations of differentially abundant metabolites and differentially abundant species between HC and DSS groups. **(B)** Associations of differentially abundant metabolites and differentially abundant species between DSS and KT2 groups. **(C)** Integration of microbiomes and metabolomes identified a UC-associated network. **(D)** Integration of microbiomes and metabolomes showing a UC + KT2 treatment-associated network. Red lines indicate positive correlations, green lines indicate negative correlations. Abundance of metabolites significant in networks provided as boxplots adjacent to relevant nodes.

To distinguish changes in metabolic and metagenomic characteristics between UC and KT2 mice, we performed integrated network analysis of microbes and metabolites (Figure 5). Results showed that the stearamide, salsolinol, wogonin (positively), and docosatrienoic acid (negatively) metabolites were associated with most species enriched in the HC group, whereas adenine, phomalone, leucylproline, histidine, and eicosapentaenoic acid were negatively associated with most species enriched in the DSS group (Figure 5A). After KT2 treatment, the prolylleucine (negatively) and salsolinol (positively) metabolites were associated with most species enriched in the KT2 group (Figure 5B). *Bacteroides* and *Lachnospiraceae* were associated with most DMs between the HC and KT2 groups, with opposite correlation relationships (Supplementary Figure S3).

We also created separate correlation networks for the differential microbes (species) and DMs (Supplementary Figure S4). Results showed that the DMs and microbes between the HC and DSS groups were highly associated, suggesting close interactions.

We performed network analysis to assess associations between the microbiome and UC-linked metabolites based on the integration of the metabolomic and metagenomic datasets as described above (Figures 5A,B). Three distinct microbiome/metabolite clusters were defined between the HC and DSS groups, including associations between *Bacteroides* and a group of 12 metabolites (three enriched and nine depleted in the DSS group), associations between *Odoribacter* and a group of 12 metabolites (two enriched and 10 depleted in the DSS group), and associations between *Anaerotruncus* and a group of 17 metabolites (two enriched and 15 depleted in the DSS group; Figure 5C). Furthermore, three distinct microbiome/metabolite clusters were defined between the DSS and KT2 groups, including associations between *Anaerotruncus* and a group of eight metabolites (four enriched and four depleted in the KT2 group), associations between *Lachnospiraceae* and a group of five metabolites (two enriched and three depleted in the KT2 group), and associations between *Burkholderiales* and a group of three metabolites (depleted in the KT2 group; Figure 5D). These results suggest that UC-associated species and metabolites could be used for testing in clinical models.

## 4. Discussion

In the current study, we examined the intestinal microbiome and metabolome of UC mice. Results revealed that the fecal microbiome and metabolome of UC mice differed significantly from those of healthy mice, and the altered microbiome and metabolome could be modified by KT2 treatment.

The levels of *Anaerotruncus* were markedly decreased in the DSS-induced UC mice compared to the HC group, consistent with previous reports (Lin et al., 2019; Shao et al., 2021). In contrast, the relative abundance of *Anaerotruncus* was enhanced after KT2 treatment, suggesting a protective role of *Anaerotruncus* in UC mice. Furthermore, DMs in the KT2-treated group were significantly enriched in the bile acid biosynthesis pathway, consistent with previous research (Shao et al., 2021). A recent study demonstrated that the symptoms of DSS-induced colitis in mice can be ameliorated by modulating intestinal microbiota, including *Anaerotruncus,* and bile acid metabolism (Huang et al., 2022). These results suggest that KT2 may improve *Anaerotruncus-induced* mucosal damage by regulating intestinal microbiota dysbiosis and bile acid metabolism. Our results also showed that *Lachnospiraceae* abundance was lower in the UC mice than in the HC mice. As *Lachnospiraceae* is reportedly unaffected by UC, decreased abundance may play a role in triggering the recurrence of UC (Sasaki et al., 2019). Dysbiosis of microbiota, including *Lachnospiraceae*, can cause dysregulation of mucosal immunity and abnormal intestinal permeability (Quraishi et al., 2017). Furthermore, high Immunoscores are associated with high *Lachnospiraceae* abundance in the microbiomes of patients with colorectal cancer (CRC), indicating an association between lymphocyte infiltration and *Lachnospiraceae* family enrichment in the gut microbiome (Hexun et al., 2022). However, although *Lachnospiraceae* is increased in certain diseases, including primary sclerosing cholangitis and IBD, many studies have shown that *Lachnospiraceae* may also influence healthy function (Vacca et al., 2020). A decrease in *Lachnospiraceae* abundance can have negative health implications as this family also performs beneficial functions (Sorbara et al., 2020). *Lachnospiraceae* can be rapidly lost after antibiotic treatment and altered by dietary changes (David et al., 2014). *Bacteroides* levels were also markedly increased in the UC mice in the present study. *Bacteroides* species are considered as enterotypes, which are enriched in the biosynthesis of different vitamins (Arumugam et al., 2011). Thus, these results suggest that attention should be paid to protecting microbial diversity in UC patients in clinical treatment, with cautious use of antibiotics.

Recent research has demonstrated that deficiency in ANG1, an intestinally secreted AMP, can protective gut commensal strains of *Lachnospiraceae* and that ANG1 can maintain gut health by promoting *Lachnospiraceae* growth (Sun et al., 2021). This is consistent with our study showing increased *Lachnospiraceae* abundance in UC mice treated with KT2. These results suggest that AMPs may be developed as a potential therapy for UC. However, further studies are needed to investigate the regulatory role of *Lachnospiraceae* in the prevention and treatment of UC.

Our results further showed that differential abundance of *Mucispirillum* substantially influenced UC outcome. Consistently, previous studies have shown that *Mucispirillum* species (e.g., *Mucispirillum schaedleri*) are protective against serovar Typhimurium-induced colitis in mice by interfering with pathogen invasion and virulence factor expression (Herp et al., 2019) and are linked with various diseases such as IBD (Herp et al., 2021). *Mucispirillum* species have also been identified as indicators of DSS-colitis in mice (Berry et al., 2012), and as biomarkers of spontaneous colitis in mouse models of IBD (Vereecke et al., 2014). Furthermore, *Mucispirillum* is reported to trigger T-cell-dependent immunoglobulin A (IgA) and immunoglobulin G (IgG) responses, suggesting that these species may exhibit efficient immune priming (Jergens et al., 2007; Bunker et al., 2015). Changes in microbes, such as *Mucispirillum*, can exacerbate intestinal inflammation, with proinflammatory microbiota influencing invariant natural killer T (iNKT) cell function upon activation during DSS colitis (Selvanantham et al., 2016). However, it has been noted in few human studies since its low relative abundance in human fecal samples. Thus, further studies on *Mucispirillum* within the human gut are necessary.

UC is charactered by severe inflammation, compromised colonic barrier, and dysbiosis of intestinal flora. Various studies have validated the role of AMPs in UC (Stebe-Frick et al., 2018; Shang et al., 2021; Liu et al., 2022), with potential involvement *via* the Toll-like receptor 4 and PI3K/Akt signaling pathways (Huang and Huang, 2018; Kim et al., 2020). KT2 is also associated with down-regulation of the PI3K/AKT/mTOR signaling pathway (Maijaroen et al., 2018). In the present study, we found that KT2 alleviated the symptoms of UC by modulating and restoring the intestinal microbiota of UC mice closer to that found in HC mice. Although we did not study the detailed mechanisms of KT2 and differential microbes in the treatment of UC, the identification of bacteria that mediate resistance to UC, such as *Anaerotruncus, Lachnospiraceae, and Mucispirillum*, is essential for studying their effects on pathogens at the functional level.

In conclusion, we identified *Anaerotruncus, Lachnospiraceae,* and *Mucispirillum* as protective species against DSS-induced UC in mice. Moreover, we established microbiome and metabolome networks in UC mice with/without KT2 treatment as additional mechanisms by which intestinal microbiota regulates bile acid biosynthesis. Our study was limited to capturing functional changes in epithelial cells after changes in microbes and KT2 treatment. However, despite this limitation, discriminatory signals were present in the microbiomes and metabolomes, supporting the gut as a potential UC biomarker. The mechanisms of action of these microbes and metabolites in UC should be further evaluated.

## Data availability statement

The datasets presented in this study can be found in online repositories. The names of the repository/repositories and accession number(s) can be found in the article/Supplementary material.

## Ethics statement

The animal study was reviewed and approved by the Animal Ethics Committee of Kunming Medical University (No. kmmu20211575).

## Author contributions

JM, YM, and JJ conceived and designed the study. QN and YY performed study subject recruitment, oversaw sample collection, metagenomic sequencing, and metabolite analysis. XJ performed hematoxylin–eosin (H&E) staining and enzymelinked immunosorbent assay. JJ and YT contributed to data analysis. JM and QN contributed to manuscript preparation. All authors reviewed the draft version and approved the final submission. All authors contributed to the article and approved the submitted version.

## Funding

This work was partly supported by the National Natural Science Foundation of China (U1802282, 82170550, and 82260107), Medicine Leading Talent of Health and Family Planning Commission of Yunnan Province (L-201607), and Yunnan Health Training Project of High Level Talents (H-2018040). The “Rejuvenating Yunnan Talents Support Plan” for Prestigious Doctors (RLMY20220010).

## Conflict of interest

The authors declare that the research was conducted in the absence of any commercial or financial relationships that could be construed as a potential conflict of interest.

## Publisher’s note

All claims expressed in this article are solely those of the authors and do not necessarily represent those of their affiliated organizations, or those of the publisher, the editors and the reviewers. Any product that may be evaluated in this article, or claim that may be made by its manufacturer, is not guaranteed or endorsed by the publisher.

## Supplementary material

The Supplementary material for this article can be found online at: https://www.frontiersin.org/articles/10.3389/fmicb.2023.1027658/full#supplementary-material

## Footnotes

^1^ https://www.metaboanalyst.ca/
