# Supplementary material for: Alterations in metabolome and microbiome signatures provide clues to the role of antimicrobial peptide KT2 in ulcerative colitis
Source: Front Microbiol. 2023 Feb 9;14:1027658. doi: 10.3389/fmicb.2023.1027658 (PMC9947474; doi:10.3389/fmicb.2023.1027658)
Supplement: Supplementary file 1 [file Data_Sheet_1.docx]

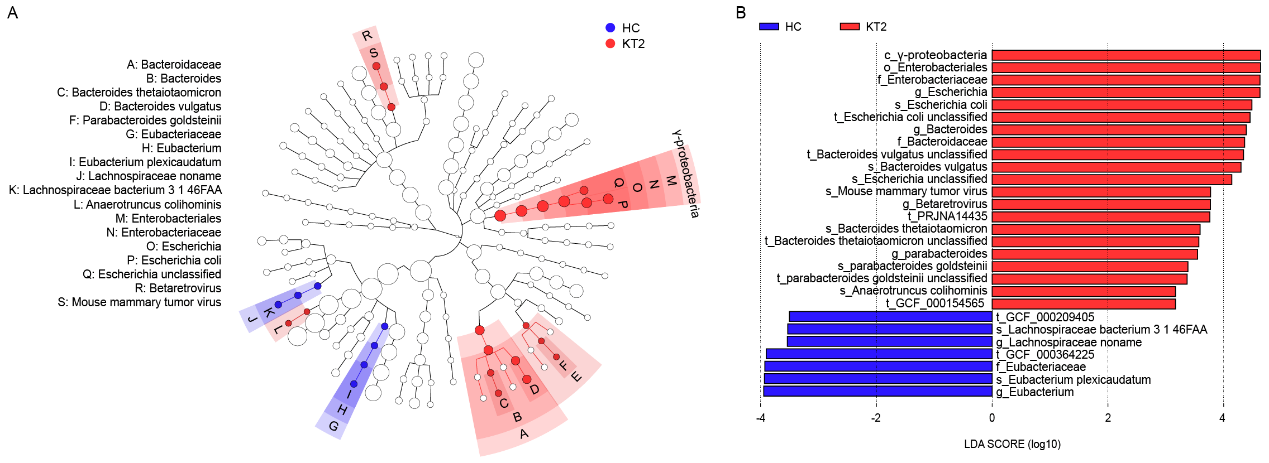


**Figure** **S1**. **Linear discriminant analysis (LDA) effect size (****LEfSe) between HC and KT2 groups.** Cladogram indicating the phylogenetic distribution of microbiota correlated with the DSS and KT2 groups.


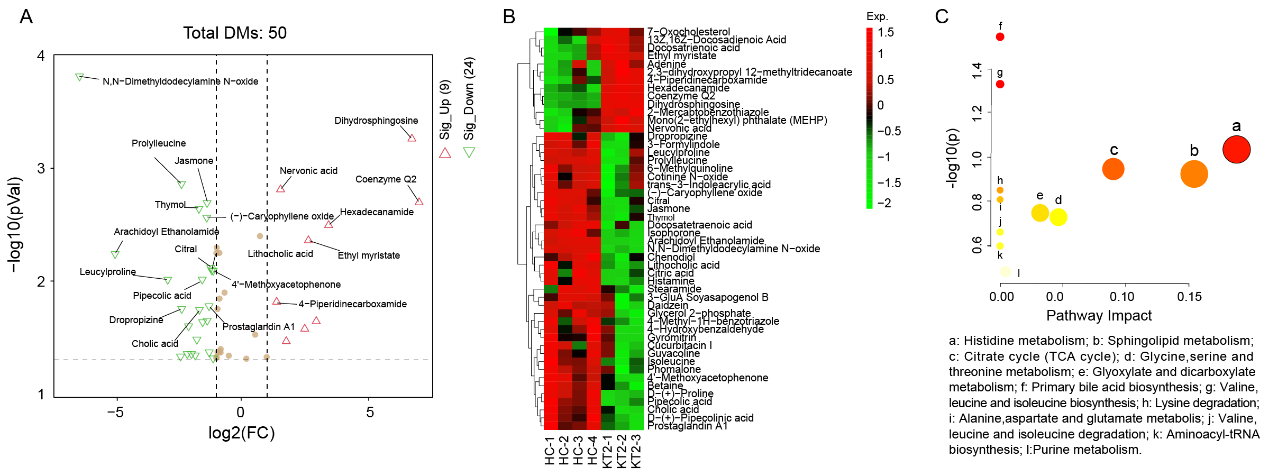


**Figure S2. Fecal metabolomics for quantification of metabolites in both HC and KT2 groups.** (**A**) Volcano plot showing differentially accumulated [log_2_(FC) on X-axis] and significantly changed [-log_10_ (*p*) on Y-axis] metabolites in the HC and KT2 groups. (**b**) Hierarchical cluster analysis of metabolites in the HC and KT2 groups based on z-normalized abundances. (**C**) Pathway enrichment and significance of DMs between the HC and KT2 groups.


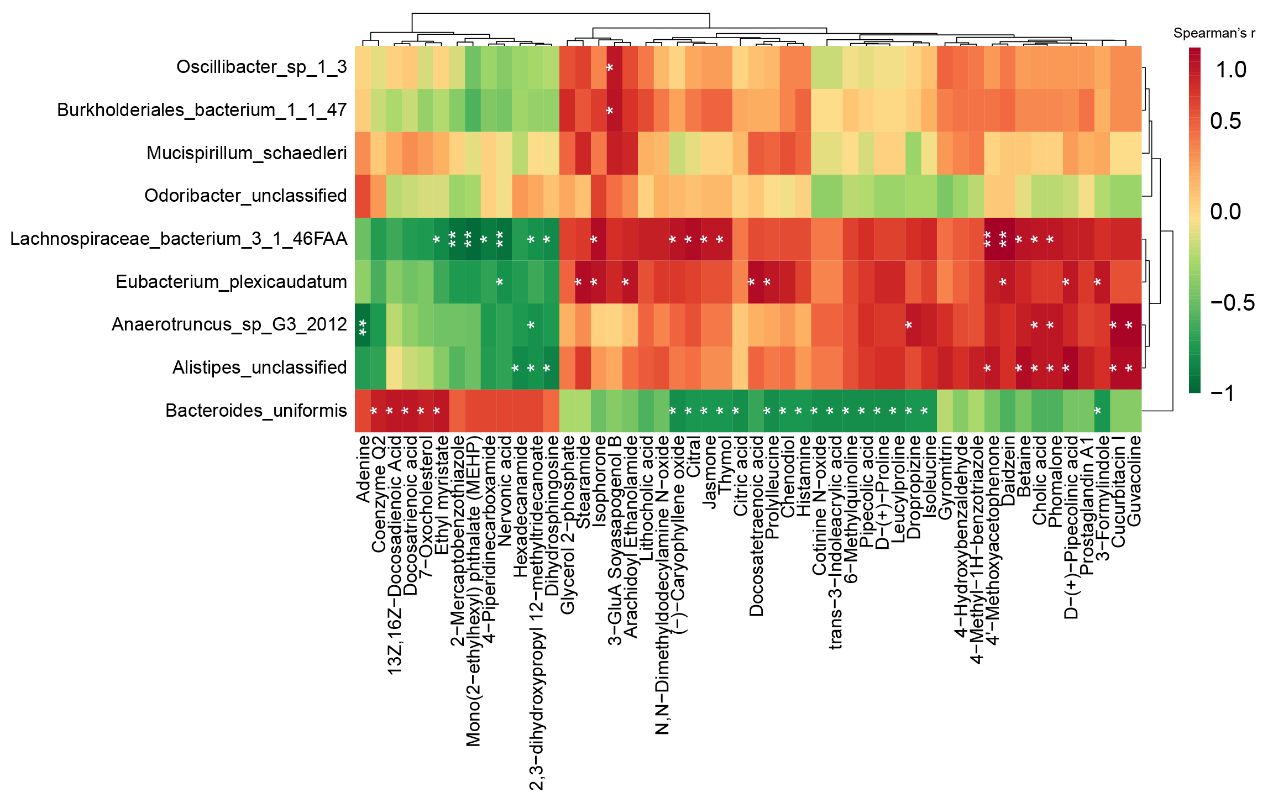


**Figure S3.** **Integration of differential microbiomes and metabolomes between HC and KT2 groups.** Red color indicates a positive correlation and green indicates a negative correlation. ^*^*p* < 0.05, ^**^*p* < 0.01.


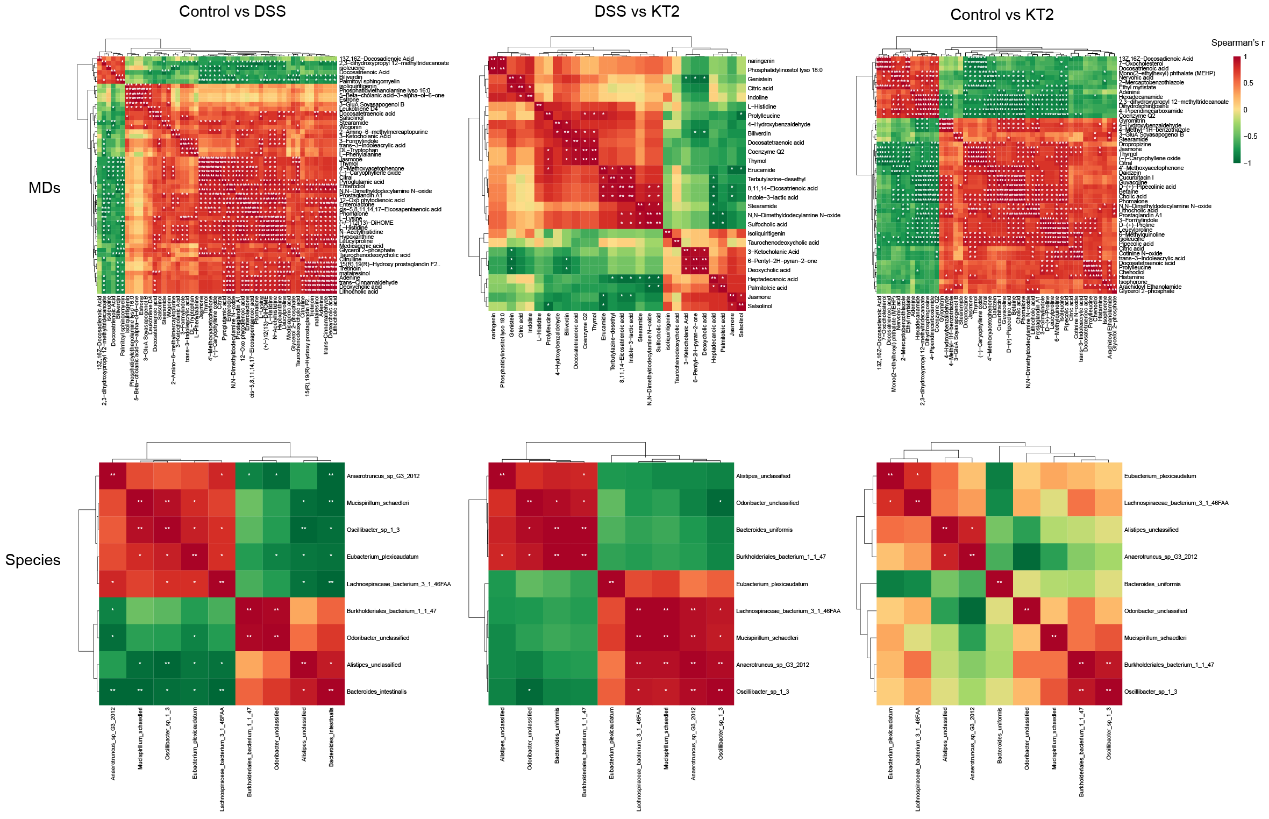


**Figure S4. Correlation network of differential microbes (species) and MDs separately.** Red color indicates a positive correlation and green indicates a negative correlation. ^*^*p* < 0.05, ^**^*p* < 0.01, ^***^*p* < 0.001.
